# Crystal structure of di-μ-hydroxido-κ^4^ *O*:*O*-bis[bis(acetyl­acetonato-κ^2^ *O*,*O*′)cobalt(III)]

**DOI:** 10.1107/S2056989015013663

**Published:** 2015-07-29

**Authors:** Casseday P. Richers, Jeffery A. Bertke, Thomas B. Rauchfuss

**Affiliations:** a School of Chemical Sciences, University of Illinois at Urbana-Champaign, Urbana, Illinois 61801, USA

**Keywords:** crystal structure, cobalt(III), acac ligand, bridging hydroxide ligand

## Abstract

The dinuclear title complex, [Co_2_(C_5_H_7_O_2_)_4_(μ-OH)_2_] or [Co(acac)_2_(μ-OH)]_2_, where acac is acetyl­acetonate, is centrosymmetric with half of the mol­ecule per asymmetric unit. The mol­ecular structure is a dimer of octa­hedrally coordinated Co^III^ atoms with four O atoms from two chelating acac ligands and two O atoms from bridging hydroxide ligands. The crystal packing features weak C—H⋯O inter­actions between neighboring mol­ecules, leading to the formation of chains normal to the *ac* plane. The hydroxide H atoms are not involved in hydrogen bonding because of the bulky acac ligands. This is the first crystal structure reported of a dimeric transition metal bis-acac complex with OH^−^ as the bridging group.

## Abstract 2

[Co(acac)_2_(μ-OH)]_2_ (acac = acetyl­acetonate) is the first crystal structure reported of a dimeric transition metal bis-acac complex with OH^−^ as the bridging group. The centrosymmetric mol­ecular structure is a [Co_2_(μ_2_-OH)_2_] dimer with each metal coordinated by two acac ligands in a κ^2^-*O*,*O*′ mode.

## Chemical context   

Well-defined cobalt(III) hydroxide complexes are relatively rare, especially in the absence of amine ligands (Bryndza & Tam, 1988 ▸). One of the earliest examples is [Co(acac)_2_(μ-OH)]_2_ (acac is acetyl­acetonate, C_5_H_7_O_2_), (I), which was prepared by oxidation of Co(II)(acac)_2_ with hydrogen peroxide. The complex reacts with 2,4-penta­nedione to form Co^III^(acac)_3_ and may serve as a useful model for hydration and oxidation catalysts (Masłowska & Baranovski, 1978 ▸; Bergquist *et al.*, 2003 ▸; Zinn *et al.*, 2007 ▸; Wang *et al.*, 2009 ▸) Boucher and Herrington characterized the complex according to IR and ^1^H NMR spectra (Boucher & Herrington, 1971 ▸). These data indicated a single diastereoisomer, the identity of which was not clear from the spectra. We now report its crystal structure, confirming that it is centrosymmetric.
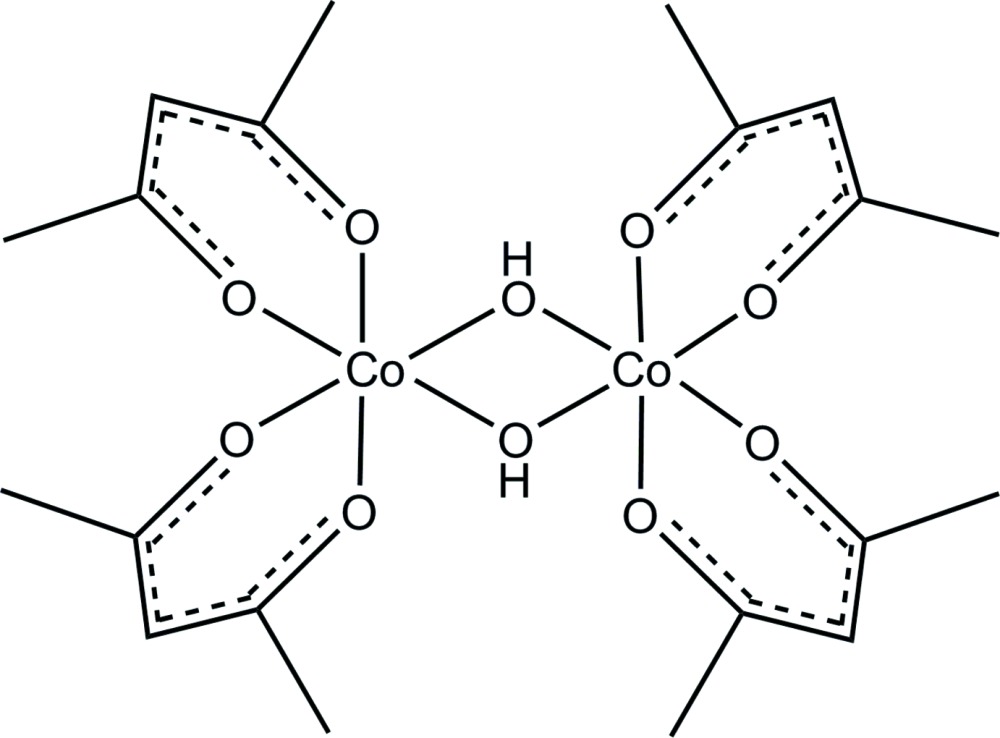


## Structural commentary   

The structure of (I) contains one crystallographically independent Co^III^ atom with an approximately octa­hedral coord­in­ation environment. The coordination sphere of Co1 is filled by the oxygen atoms of two κ^2^-*O*,*O*′ acac ligands [Co1—O2 = 1.8830 (16) Å, Co1—O3 = 1.8770 (16) Å, Co1—O4 = 1.8814 (16) Å, Co1—O5 = 1.8820 (17) Å) and two μ_2_-hydroxyl groups [Co1—O1 = 1.9131 (16) Å, Co1—O1^i^ = 1.9087 (17) Å; symmetry code: (i) −x, −y+2, −z+1]. The angles around Co1 are distorted slightly from the ideal 90° and 180° of a perfect octa­hedron. The *cis* angles range from 82.07 (7) to 95.92 (7)° while the *trans* angles range from 173.53 (7) to 178.37 (6)°.

**Figure 1:**
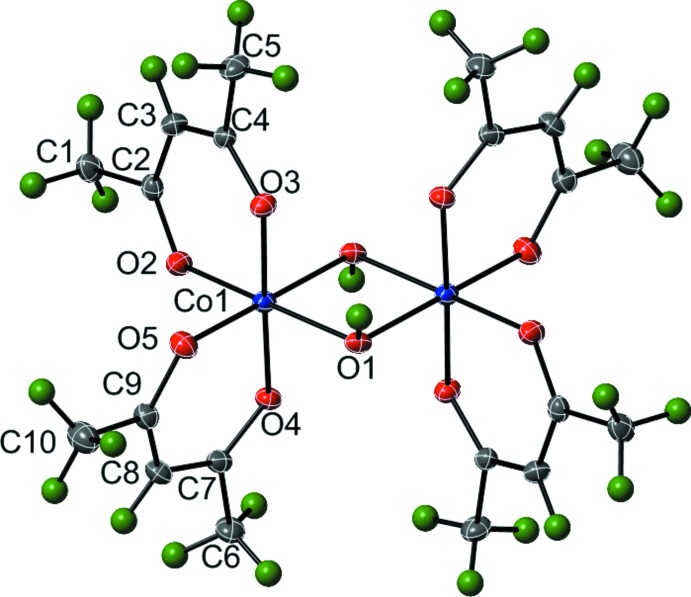
The mol­ecular structure of (I), showing displacement ellipsoids at the 35% probability for non-H atoms and spheres of arbitrary size for H atoms. The unlabeled atoms are related by the symmetry operator (−*x*, −*y* + 2, −*z* + 1).

The mol­ecular structure of (I) contains a [Co_2_(μ_2_-OH)_2_] motif with each metal coordinated by two acac ligands in a κ^2^-*O*,*O*′ mode (Fig. 1 ▸). The two halves of the dimer are related *via* inversion symmetry. The Co1⋯Co1^i^ distance is 2.8829 (7) Å. This distance falls within the range (2.696–3.355 Å) of all Co⋯Co distances reported in the Cambridge Crystallographic Database (Groom & Allen, 2014 ▸) for OH^−^-bridged Co complexes in which the metals are coordinated by six oxygen atoms. It is well below the average Co⋯Co distance of 3.108 Å.

## Supra­molecular features   

**Figure 2:**
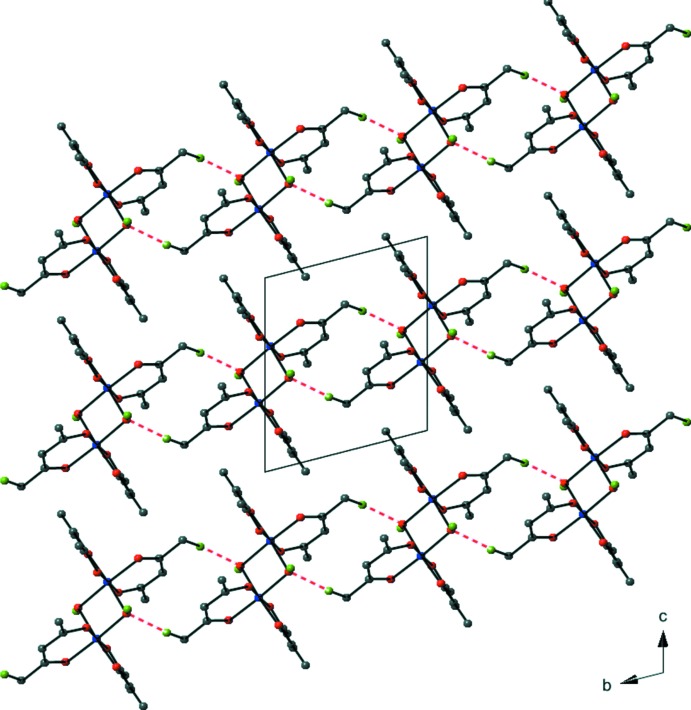
A view along the *a* axis of the crystal structure of (I), showing extended chains normal to the *ac* plane. The weak C—H⋯O inter­actions are shown as red dashed lines. All H atoms except the hydroxide H atom (H1) and the inter­acting H atoms (H1*B*) have been omitted for clarity. Color code: blue = Co, red = O, gray = C, green = H.

There are no significant supra­molecular features to discuss with the extended structure of (I). There are weak C—H⋯O inter­molecular inter­actions (Table 1 ▸) between one methyl group of an acac ligand and the hydroxide oxygen atom. These inter­actions result in the formation of chains normal to the *ac* plane (Fig. 2 ▸). It should be noted that the hydroxyl H atom does not participate in hydrogen bonding. Examination of the packing diagram shows that the bulky acac ligands prevent any hydrogen-bonding inter­actions with neighboring mol­ecules.

## Database survey   

One closely related crystal structure, [Co(*L*)_2_(μ-OH)]_2_; *L* = 1-(di­benzyl­amino)-5,5-dimethyl-1,4-dioxohex-2-en-2-olate, has been reported previously (Wang *et al.*, 2009 ▸). The ligand in this complex is a modified acac with a *tert*-butyl group in place of one methyl and a {CON(CH_2_Ph)_2_} group in place of the other methyl group. The coordination environment of the Co^III^ atoms is the same as in (I). The average Co—O_*L*_ distance of 1.890 Å is similar to the average Co—O_acac_ distance in (I) of 1.881 Å. The average Co—OH distance of 1.907 Å is also comparable to that of (I) (1.911 Å).

A search of the Cambridge Crystallographic Database (Groom & Allen, 2014 ▸) returned 13 dimeric complexes with the general formula [*TM*(acac)_2_(μ-*X*)]_2_; *TM* = transition metal, and *X* = O, O*R*, NO, or S (Bottomley *et al.*, 1982 ▸; Nakahanada *et al.*, 1992 ▸; Smith *et al.*, 1972 ▸; Sokolov *et al.*, 1999 ▸). Complex (I) is the first crystal structure reported that fits this general formula in which the bridging group is OH^−^.

## Synthesis and crystallization   

The title complex was synthesized according to the procedures reported by Boucher & Herrington (1971 ▸). To a mixture of Co(acac)_2_·2H_2_O (2 g, 7.27×10 ^−3^ mol, 1 equiv) and KOAc (3.2 g, 3.26×10 ^−2^ mol, 4.5 equiv) in methanol (125 ml) was added a solution of H_2_O_2_ in water (30%_wt_, 2 ml). The resulting solution changed color from pink to green. The reaction was stirred at room temperature for 1 h under an ambient atmos­phere. The reaction was then concentrated to dryness on a rotary evaporator. The residual green solid was washed with water (3 × 20 ml) and then acetone (3 × 20 ml), and then dried in air, leaving the product (0.85 g, 1.55×10 ^−3^ mol, 43% yield). Crystals, suitable for X-ray diffraction, were grown by slow diffusion of pentane into chloro­form solutions of the green product.

## Refinement   

Crystal data, data collection and structure refinement details are summarized in Table 2 ▸. The hydroxyl H atom was located in a difference map and its position was allowed to refine freely. Methyl H atom positions, *R*-CH_3_, were optimized by rotation about *R*—C bonds with idealized C—H, *R*—H and H⋯H distances. Remaining H atoms were included as riding idealized contributors. Methyl and hydroxide H atom *U*_iso_’s were assigned as 1.5*U*_eq_ of the carrier atom; remaining H atom *U*_iso_’s were assigned as 1.2*U*_eq_ of the carrier atom.

## Supplementary Material

Crystal structure: contains datablock(s) I. DOI: 10.1107/S2056989015013663/wm5186sup1.cif

Structure factors: contains datablock(s) I. DOI: 10.1107/S2056989015013663/wm5186Isup2.hkl

Supporting information file. DOI: 10.1107/S2056989015013663/wm5186Isup3.cdx

CCDC reference: 1413567

Additional supporting information:  crystallographic information; 3D view; checkCIF report

## Figures and Tables

**Table 1:** Hydrogen-bond geometry (, )

*D*H*A*	*D*H	H*A*	*D* *A*	*D*H*A*
C1H1*B*O1^i^	0.98	2.42	3.395(3)	174

Symmetry code: (i) 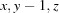.

**Table 2:** Experimental details

Crystal data	
Chemical formula	[Co_2_(C_5_H_7_O_2_)_4_(OH)_2_]
*M*_r_	548.30
Crystal system, space group	Triclinic, *P* 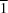
Temperature (K)	173
*a*, *b*, *c* ()	7.8610(11), 8.2481(11), 9.8372(13)
, , ()	100.786(8), 106.708(8), 99.492(9)
*V* (^3^)	583.67(14)
*Z*	1
Radiation type	Mo *K*
(mm^1^)	1.47
Crystal size (mm)	0.23 0.19 0.04

Data collection	
Diffractometer	Bruker Kappa APEXII CCD
Absorption correction	Integration (*SADABS*; Bruker, 2012)
*T*_min_, *T*_max_	0.776, 0.945
No. of measured, independent and observed [*I* > 2(*I*)] reflections	16164, 2617, 2228
*R*_int_	0.083
(sin /)_max_ (^1^)	0.646

Refinement	
*R*[*F* ^2^ > 2(*F* ^2^)], *wR*(*F* ^2^), *S*	0.036, 0.093, 1.05
No. of reflections	2617
No. of parameters	152
H-atom treatment	H atoms treated by a mixture of independent and constrained refinement
_max_, _min_ (e ^3^)	0.39, 0.53

Computer programs: *APEX2* and *SAINT* (Bruker, 2013 ▸), *SHELXS* and *SHELXTL* (Sheldrick, 2008 ▸), *SHELXL2013*/4 (Sheldrick, 2015 ▸), *CrystalMaker* (CrystalMaker, 1994 ▸), *XCIF* (Bruker, 2013 ▸) and *publCIF* (Westrip, 2010 ▸).

## supplementary crystallographic information

### Crystal data

**Table:**

[Co_2_(C_5_H_7_O_2_)_4_(OH)_2_]	*Z* = 1
*M**_r_* = 548.30	*F*(000) = 284
Triclinic, *P*1	*D*_x_ = 1.560 Mg m^−^^3^
*a* = 7.8610 (11) Å	Mo *K*α radiation, λ = 0.71073 Å
*b* = 8.2481 (11) Å	Cell parameters from 3791 reflections
*c* = 9.8372 (13) Å	θ = 2.6–25.5°
α = 100.786 (8)°	µ = 1.47 mm^−^^1^
β = 106.708 (8)°	*T* = 173 K
γ = 99.492 (9)°	Plate, blue
*V* = 583.67 (14) Å^3^	0.23 × 0.19 × 0.04 mm

### Data collection

**Table:**

Bruker Kappa APEXII CCD diffractometer	2617 independent reflections
Radiation source: fine-focus sealed tube	2228 reflections with *I* > 2σ(*I*)
Graphite monochromator	*R*_int_ = 0.083
profile data from φ and ω scans	θ_max_ = 27.3°, θ_min_ = 2.2°
Absorption correction: integration (*SADABS*; Bruker, 2012)	*h* = −10→10
*T*_min_ = 0.776, *T*_max_ = 0.945	*k* = −10→10
16164 measured reflections	*l* = −12→12

### Refinement

**Table:**

Refinement on *F*^2^	0 restraints
Least-squares matrix: full	Hydrogen site location: mixed
*R*[*F*^2^ > 2σ(*F*^2^)] = 0.036	H atoms treated by a mixture of independent and constrained refinement
*wR*(*F*^2^) = 0.093	*w* = 1/[σ^2^(*F*_o_^2^) + (0.0366*P*)^2^ + 0.1984*P*] where *P* = (*F*_o_^2^ + 2*F*_c_^2^)/3
*S* = 1.05	(Δ/σ)_max_ = 0.001
2617 reflections	Δρ_max_ = 0.39 e Å^−^^3^
152 parameters	Δρ_min_ = −0.53 e Å^−^^3^

### Special details

**Table:**

Experimental. One distinct cell was identified using *APEX2* (Bruker, 2013). Fourteen frame series were integrated and filtered for statistical outliers using *SAINT* (Bruker, 2013) then corrected for absorption by integration using *SAINT*/*SADABS*, v2012/1 (Bruker, 2012) to sort, merge, and scale the combined data. No decay correction was applied.
Geometry. All e.s.d.'s (except the e.s.d. in the dihedral angle between two l.s. planes) are estimated using the full covariance matrix. The cell e.s.d.'s are taken into account individually in the estimation of e.s.d.'s in distances, angles and torsion angles; correlations between e.s.d.'s in cell parameters are only used when they are defined by crystal symmetry. An approximate (isotropic) treatment of cell e.s.d.'s is used for estimating e.s.d.'s involving l.s. planes.
Refinement. Structure was phased by direct methods (Sheldrick, 2008). Systematic conditions suggested the ambiguous space group. The space group choice was confirmed by successful convergence of the full-matrix least-squares refinement on *F*^2^. The final map had no significant features. A final analysis of variance between observed and calculated structure factors showed little dependence on amplitude and resolution.

### Fractional atomic coordinates and isotropic or equivalent isotropic displacement parameters (Å^2^)

**Table:**

	*x*	*y*	*z*	*U*_iso_*/*U*_eq_	
Co1	0.08356 (4)	0.96491 (4)	0.63894 (3)	0.01839 (12)	
O1	0.0955 (2)	1.1492 (2)	0.54669 (18)	0.0197 (4)	
H1	0.182 (4)	1.158 (4)	0.530 (3)	0.030*	
O2	0.0442 (2)	0.7772 (2)	0.71880 (18)	0.0244 (4)	
O3	0.2682 (2)	0.9024 (2)	0.56955 (18)	0.0220 (4)	
C1	0.0467 (4)	0.5042 (3)	0.7559 (3)	0.0294 (6)	
H1A	0.1238	0.5263	0.8584	0.044*	
H1B	0.0576	0.3969	0.7002	0.044*	
H1C	−0.0808	0.4967	0.7508	0.044*	
C2	0.1074 (3)	0.6460 (3)	0.6920 (2)	0.0216 (5)	
C3	0.2293 (3)	0.6301 (3)	0.6154 (3)	0.0239 (5)	
H3	0.2605	0.5236	0.5962	0.029*	
C4	0.3092 (3)	0.7594 (3)	0.5647 (2)	0.0204 (5)	
C5	0.4588 (3)	0.7360 (3)	0.5010 (3)	0.0291 (6)	
H5A	0.4703	0.8171	0.4411	0.044*	
H5B	0.4290	0.6201	0.4398	0.044*	
H5C	0.5745	0.7556	0.5805	0.044*	
O4	−0.1041 (2)	1.0298 (2)	0.70365 (18)	0.0211 (3)	
O5	0.2764 (2)	1.0871 (2)	0.81009 (18)	0.0258 (4)	
C6	−0.2501 (3)	1.1417 (4)	0.8641 (3)	0.0302 (6)	
H6A	−0.2682	1.2526	0.8490	0.045*	
H6B	−0.2371	1.1397	0.9658	0.045*	
H6C	−0.3556	1.0527	0.7980	0.045*	
C7	−0.0812 (3)	1.1114 (3)	0.8328 (3)	0.0216 (5)	
C8	0.0873 (3)	1.1769 (3)	0.9434 (3)	0.0265 (5)	
H8	0.0877	1.2323	1.0373	0.032*	
C9	0.2534 (3)	1.1666 (3)	0.9258 (3)	0.0245 (5)	
C10	0.4268 (4)	1.2530 (4)	1.0500 (3)	0.0383 (7)	
H10A	0.4992	1.1698	1.0733	0.057*	
H10B	0.3972	1.3033	1.1363	0.057*	
H10C	0.4973	1.3424	1.0215	0.057*	

### Atomic displacement parameters (Å^2^)

**Table:**

	*U*^11^	*U*^22^	*U*^33^	*U*^12^	*U*^13^	*U*^23^
Co1	0.01960 (18)	0.01881 (19)	0.02005 (19)	0.00636 (13)	0.01007 (13)	0.00540 (13)
O1	0.0196 (8)	0.0184 (8)	0.0237 (9)	0.0052 (7)	0.0118 (7)	0.0033 (7)
O2	0.0309 (9)	0.0253 (9)	0.0253 (9)	0.0113 (7)	0.0164 (7)	0.0102 (7)
O3	0.0223 (8)	0.0223 (9)	0.0267 (9)	0.0086 (7)	0.0130 (7)	0.0083 (7)
C1	0.0407 (15)	0.0259 (13)	0.0245 (13)	0.0060 (11)	0.0144 (11)	0.0090 (10)
C2	0.0227 (12)	0.0217 (12)	0.0162 (11)	0.0041 (9)	0.0015 (9)	0.0035 (9)
C3	0.0276 (12)	0.0198 (12)	0.0253 (13)	0.0091 (10)	0.0091 (10)	0.0041 (10)
C4	0.0199 (11)	0.0214 (12)	0.0166 (11)	0.0057 (9)	0.0023 (9)	0.0018 (9)
C5	0.0292 (13)	0.0297 (14)	0.0366 (15)	0.0149 (11)	0.0175 (11)	0.0099 (11)
O4	0.0210 (8)	0.0233 (9)	0.0218 (8)	0.0066 (7)	0.0114 (7)	0.0042 (7)
O5	0.0216 (9)	0.0323 (10)	0.0231 (9)	0.0069 (7)	0.0088 (7)	0.0033 (7)
C6	0.0301 (13)	0.0399 (15)	0.0278 (14)	0.0147 (12)	0.0167 (11)	0.0085 (12)
C7	0.0292 (12)	0.0188 (12)	0.0231 (12)	0.0075 (10)	0.0155 (10)	0.0080 (9)
C8	0.0317 (13)	0.0303 (14)	0.0196 (12)	0.0094 (11)	0.0120 (10)	0.0034 (10)
C9	0.0282 (13)	0.0260 (13)	0.0214 (12)	0.0074 (10)	0.0098 (10)	0.0074 (10)
C10	0.0313 (14)	0.0479 (18)	0.0277 (15)	0.0049 (13)	0.0074 (12)	−0.0021 (13)

### Geometric parameters (Å, º)

**Table:**

Co1—O3	1.8770 (16)	C4—C5	1.506 (3)
Co1—O4	1.8814 (16)	C5—H5A	0.9800
Co1—O5	1.8820 (17)	C5—H5B	0.9800
Co1—O2	1.8830 (16)	C5—H5C	0.9800
Co1—O1^i^	1.9087 (17)	O4—C7	1.268 (3)
Co1—O1	1.9131 (16)	O5—C9	1.279 (3)
Co1—Co1^i^	2.8829 (7)	C6—C7	1.495 (3)
O1—Co1^i^	1.9087 (17)	C6—H6A	0.9800
O1—H1	0.74 (3)	C6—H6B	0.9800
O2—C2	1.278 (3)	C6—H6C	0.9800
O3—C4	1.269 (3)	C7—C8	1.395 (3)
C1—C2	1.502 (3)	C8—C9	1.380 (3)
C1—H1A	0.9800	C8—H8	0.9500
C1—H1B	0.9800	C9—C10	1.502 (3)
C1—H1C	0.9800	C10—H10A	0.9800
C2—C3	1.387 (3)	C10—H10B	0.9800
C3—C4	1.393 (3)	C10—H10C	0.9800
C3—H3	0.9500		
			
O3—Co1—O4	178.37 (6)	C2—C3—C4	124.5 (2)
O3—Co1—O5	85.06 (7)	C2—C3—H3	117.8
O4—Co1—O5	95.92 (7)	C4—C3—H3	117.8
O3—Co1—O2	95.73 (7)	O3—C4—C3	125.0 (2)
O4—Co1—O2	85.55 (7)	O3—C4—C5	115.1 (2)
O5—Co1—O2	91.96 (7)	C3—C4—C5	120.0 (2)
O3—Co1—O1^i^	90.26 (7)	C4—C5—H5A	109.5
O4—Co1—O1^i^	88.67 (7)	C4—C5—H5B	109.5
O5—Co1—O1^i^	173.53 (7)	H5A—C5—H5B	109.5
O2—Co1—O1^i^	92.95 (7)	C4—C5—H5C	109.5
O3—Co1—O1	88.10 (7)	H5A—C5—H5C	109.5
O4—Co1—O1	90.54 (7)	H5B—C5—H5C	109.5
O5—Co1—O1	93.29 (7)	C7—O4—Co1	124.45 (16)
O2—Co1—O1	173.75 (7)	C9—O5—Co1	123.82 (16)
O1^i^—Co1—O1	82.07 (7)	C7—C6—H6A	109.5
O3—Co1—Co1^i^	88.91 (5)	C7—C6—H6B	109.5
O4—Co1—Co1^i^	89.47 (5)	H6A—C6—H6B	109.5
O5—Co1—Co1^i^	134.10 (5)	C7—C6—H6C	109.5
O2—Co1—Co1^i^	133.93 (6)	H6A—C6—H6C	109.5
O1^i^—Co1—Co1^i^	41.09 (5)	H6B—C6—H6C	109.5
O1—Co1—Co1^i^	40.98 (5)	O4—C7—C8	125.1 (2)
Co1^i^—O1—Co1	97.93 (7)	O4—C7—C6	116.0 (2)
Co1^i^—O1—H1	103 (2)	C8—C7—C6	118.9 (2)
Co1—O1—H1	106 (2)	C9—C8—C7	124.4 (2)
C2—O2—Co1	123.65 (15)	C9—C8—H8	117.8
C4—O3—Co1	124.41 (15)	C7—C8—H8	117.8
C2—C1—H1A	109.5	O5—C9—C8	125.7 (2)
C2—C1—H1B	109.5	O5—C9—C10	114.7 (2)
H1A—C1—H1B	109.5	C8—C9—C10	119.6 (2)
C2—C1—H1C	109.5	C9—C10—H10A	109.5
H1A—C1—H1C	109.5	C9—C10—H10B	109.5
H1B—C1—H1C	109.5	H10A—C10—H10B	109.5
O2—C2—C3	125.1 (2)	C9—C10—H10C	109.5
O2—C2—C1	115.2 (2)	H10A—C10—H10C	109.5
C3—C2—C1	119.6 (2)	H10B—C10—H10C	109.5

Symmetry code: (i) −*x*, −*y*+2, −*z*+1.

### Hydrogen-bond geometry (Å, º)

**Table:**

*D*—H···*A*	*D*—H	H···*A*	*D*···*A*	*D*—H···*A*
C1—H1*B*···O1^ii^	0.98	2.42	3.395 (3)	174

Symmetry code: (ii) *x*, *y*−1, *z*.
